# Insecticidal Activities of Diterpene Alkaloids in Plants of the Genera *Aconitum* and *Delphinium*

**DOI:** 10.3390/toxins17050254

**Published:** 2025-05-20

**Authors:** Jinqiu Wang, Luchuan Zheng, Wenxi Huang, Linxuan Li, Jialian Yuan, Lin Chen

**Affiliations:** 1 School of Life Science and Engineering, Southwest Jiaotong University, Chengdu 610031, China; 2022113463@my.swjtu.edu.cn (J.W.); 2022113454@my.swjtu.edu.cn (L.Z.); 2022113610@my.swjtu.edu.cn (W.H.); llx1551@my.swjtu.edu.cn (L.L.); 2023113592@my.swjtu.edu.cn (J.Y.); 2 School of Life Science and Engineering, Yibin Institute of Southwest Jiaotong University, Yibin 644000, China

**Keywords:** antifeedant activity, diterpenoid alkaloids, environmentally friendly, insecticidal activity, plant-derived pesticides

## Abstract

As the global population grows, food security and agricultural productivity face challenges, and insect pests cause significant losses to crops. The effectiveness of traditional chemical pesticides is declining, and eco-friendly pesticides need to be developed. Diterpenoid alkaloids (DAs), natural products of plant origin, have attracted attention due to their low environmental risks. Here we review the classification, structure, insecticidal and anti-feeding activities of diterpenoid alkaloids, as well as the current state of research on these chemicals. Studies have shown that C_19_- and C_20_-diterpenoid alkaloids show significant activity against a variety of insects, but there are still limited studies on C_18_-diterpenoid alkaloids. Therefore, through in-depth research on diterpenoid alkaloids, we have discovered that there are various compounds with high efficiency and specificity in insecticidal and antifeedant activities among C_19_- and C_20_-diterpenoid alkaloids, which exhibit high selectivity and efficiency towards target pests. This paper emphasizes the potential of diterpenoid alkaloids as novel biopesticides and highlights the need to combine new technologies to conduct further systematic evaluation and screening of these compounds. This work provides new ideas for the development of environmentally friendly pesticides and contributes to sustainable agricultural practices.

## 1. Introduction

Humanity faces many problems that arise from the rapidly increasing population, and the most important issue is the provision of the population with good quality food that is accessible for all [1]. Insect pests and mites, especially in the developing countries, are a significant cause of agricultural and forest crop losses and destroy approximately 20% of world cereal crops. Many strategies, including chemical insecticides, push−pull strategies, natural enemies, and pathogenic microbes, have been exploited to control pests, increase crop yield, and improve food production. However, the control of insect populations has become increasingly difficult because of the reduced effectiveness of pesticides caused by the emergence of resistance and rapid reproduction in arthropod pests [2]. Moreover, with the increasing pressure to provide safe food and the increasingly stringent regulatory requirements of food production, there is a critical need to discover and develop more environmentally benign and less hazardous pesticides [3].

In recent years, natural products, especially materials originating from plants and their derivatives, which are also called secondary plant metabolites [4], have become the preferred green choice due to their low risk to the environment and safety toward non-target organisms. These chemical compounds produced by plants, such as alkaloids, flavonoids, steroids, terpenoids, organic acids, and alcohols, perform useful functions against insects serving as repellents, feeding inhibitors, and toxins [5]. Pesticides of plant origin are gaining increased attention and interest among those concerned with environmentally friendly, safe, and integrated crop management approaches. In addition, they are playing a vital role in organic food production globally.

Diterpenoid alkaloids (DAs), well-known toxic plant secondary metabolites, are the characteristic components of the plant species of the genera *Aconitum* and *Delphinium* (Ranunculaceae), which are widely distributed in the temperate regions of the northern hemisphere. They have a high degree of structural diversity, which is essential for understanding the relationship between chemical structure and biological activity. These DAs in *Aconitum* and *Delphinium* constitute a structurally diverse class of biologically active natural products with a long history of being used as medicines, poisons, and insecticides, and have long attracted considerable interest, making them ideal candidates for further exploration of pesticide development. Accordingly, a great amount of research was conducted in relation to their phytochemistry, synthesis, and bioactivities. The existing body of knowledge provides a rich background for exploring their potential as a novel pesticide.

Despite extensive research on the insecticidal and anti-feeding activities of DAs, there is still a significant research gap. Firstly, while the C_19_ and C_20_ types of DAs have been widely studied, there is little research on the C_18_ type, and the bioactivities of bis-diterpenoid alkaloids have not been explored, which may have unique interactions with insect targets. Furthermore, existing studies often focus on a limited number of insect species, neglecting the possibility of species-specific responses, which restricts the broad applicability of the findings. Current research also lacks systematic comparisons of structurally diverse DAs, as most investigations are centered on a few alkaloids isolated from specific plants, resulting in an ambiguous activity–structure relationship.

In view of the fact that there is no systematic review of the biopesticide activities of diterpenoid alkaloids published previously, this review will systematically focus on the progress of diterpenoid alkaloids with different structures derived from *Aconitum* and *Delphinium* plants and some of their derivatives (e.g., lappaconitine, aconitine, songorine, pseudokobusine, and 11-veratroylpseudokobusine) with potential insecticidal and antifeedant activities. The abundance of DAs in many plants is extremely low, and traditional bioassays that rely on live insects also suffer from low throughput and resource-intensive problems, which greatly hinders large-scale activity screening. This review aims to help researchers further analyze the structure–activity relationship of DAs. Through the use of computer-aided design and other advanced technical means, the compounds with potential biological activity can be predicted, so as to reduce the risk and cost in the traditional experimental screening process and improve the efficiency of drug research and development. Additionally, the structural modification of DAs through chemical synthesis or semi-synthesis strategies is expected to optimize their pharmacological activity and provide a reference for subsequent scientific research and the development of new pesticides.

## 2. Diterpenoid Alkaloids (DAs) and Pest Control

### 2.1. Classification and Structure of DAs

**Figure 1:**
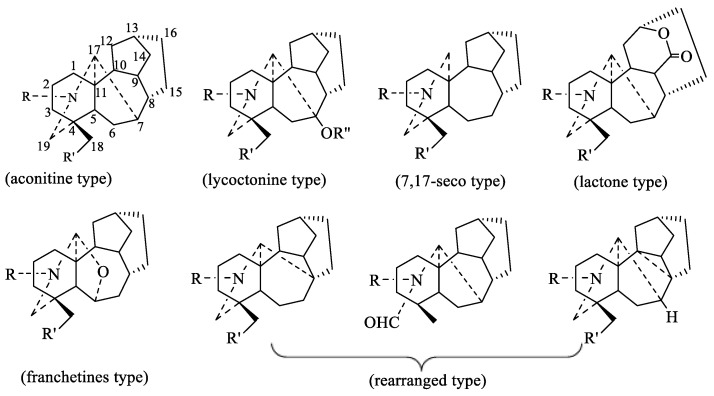
Classification of C_19_-diterpenoid alkaloids [6].

**Figure 2:**
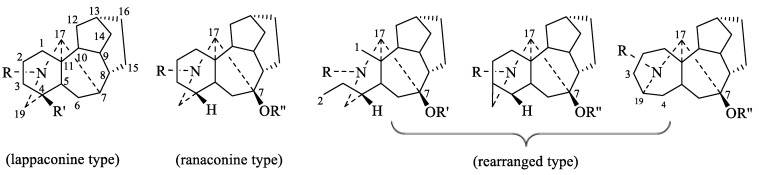
Classification of C_18_-diterpenoid alkaloids [6].

**Figure 3:**
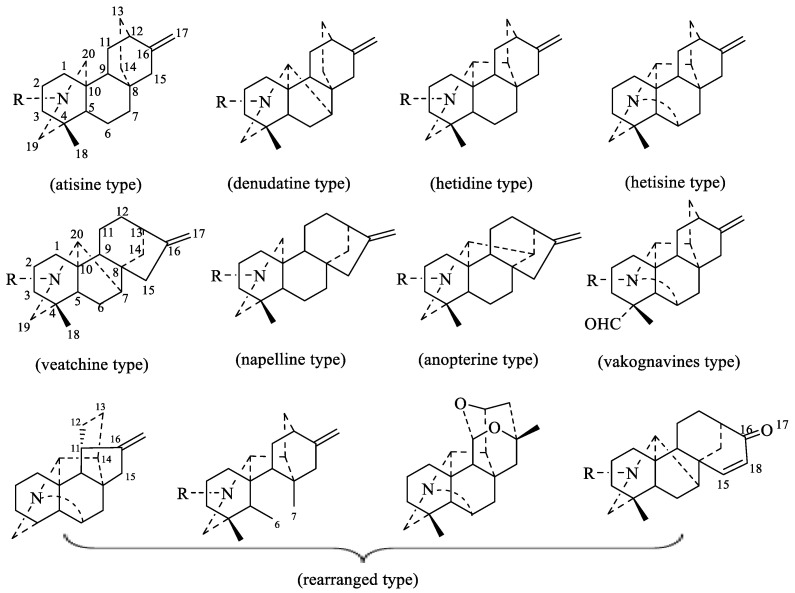
Classification of C_20_-diterpenoid alkaloids [6].

**Figure 4:**
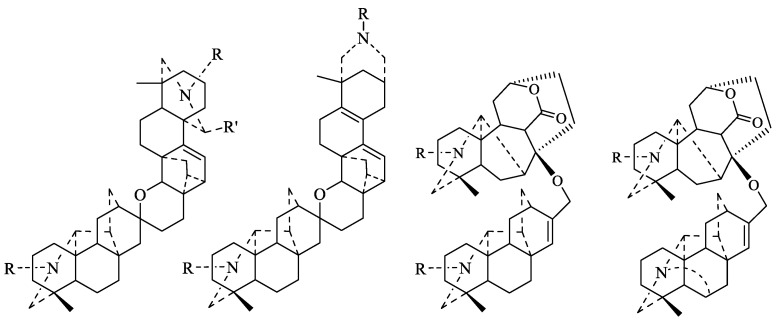
Classification of bis-diterpenoid alkaloids [6].

From the viewpoint of their biogenesis, diterpenoid alkaloids are derived from the amination of tetracyclic or pentacyclic diterpenoids in nature. As far as we know, more than 1500 natural diterpenoid alkaloids have been reported to date [6]. In 2020, Zhao and coworkers wrote a review on the structural diversity, bioactivities, and biosynthesis of natural DAs from 2009 to 2018, in which DAs were divided into four categories, C_18_-, C_19_-, C_20_-, and bis-diterpenoid categories [7]. The largest group is C_19_-diterpenoid alkaloids (Figure 1), which generally have multiple oxygen-containing substitutions and can be further divided into aconitine-type, lycoctonine-type, seco-type, and lactone-type, based on the carbon skeleton and substituents at specific positions. The C_18_-diterpenoid alkaloids, which constitute a small group within the diterpenoid alkaloids, can be divided into lappaconine-type and ranaconine-type according to the presence of oxygen-containing substitution at the C-7 position (Figure 2). C_20_-diterpenoid alkaloids have many kinds, and their molecular skeleton is composed of 20 carbon atoms, which generally contain exocyclic double bonds (Figure 3). The skeletal types of the C_20_-diterpenoid alkaloids are extremely complex compared with those of C_18_- and C_19_-diterpenoid alkaloids [8]. Bis-diterpenoid alkaloids are formed by condensation of two C_20_-diterpenoid alkaloids, or of one each of the C_19_- and C_20_- diterpenoid alkaloids (Figure 4).

### 2.2. Insecticidal and Antifeedant Activities of DAs

As indicated previously, the diterpenoid alkaloids isolated from the genera of *Delphinium* and *Aconitum* (Ranunculaceae) have been of interest because of their pharmacological properties, complex structures, and interesting chemistry since the early 1800’s. The following is a description of the research progress on the insecticidal and antifeedant activities of diterpenoid alkaloids.

#### 2.2.1. Insecticidal and Inhibiting Activities of C_19_-Ditepenoid Alkaloids

The extract from genera *Delphinium* and *Aconitum* has been used as an insecticide for a long history. As early as 1973, methyllycaconitine (MLA, **1**) was identified by Mats, using paper and thin-layer chromatography from *D. grandiflorum* L., *D. triste* Fisch, and *D. crassifolium* Schrad [9]. However, it was not until 1986 that Jennings et al. [10]. found that chloroform extracts of the seeds of *Delphinium* plant exhibit a very potent insecticidal and antifeedant activity against several species of insects and mites, such as *Spodoptera eridania*, *Heliothis virescens,* and *Musca domestiea*. Based on activity tracking, they isolated and identified the active compound MLA. Interestingly, MLA exhibited a very rapid lethal effect on *S. eridania* within 24 h, when the concentration of MLA was as low as 100 ppm. The surviving insects stopped feeding, and their growth and development were also inhibited. The lethal concentration (LC_50_) of MLA to *S. eridania* was 308 ± 48 ppm (SE), while the LC_50_ of nicotine (postsynaptic nAChRs agonist) was as high as 1000 ppm under the same experimental conditions. Furthermore, its mode of action may be associated with its high affinity for the insect nicotinic cholinergic receptor, but the activity is clearly different from that reported for the rat diaphragm muscle nicotinic receptor, which indicates a significant difference in pharmacology of the binding site between mammalian muscle and insect nerve tissue. The authors thought that the inhibition rate of nicotine on [^3^H]-propionyl-α-bungaroto-xin (^3^H α-BGTx) binding to musca domestica head homogenate (an insect nicotinic acetylcholine receptor antagonist) (Kinh = 8.2 ± 0.6 μM) was about 10,000 times smaller than that on MLA (**1**) (Kinh = 2.5 × 10^−10^ ± 0.5 × 10^−10^ M). This may be the reason contributing to the great difference in insecticidal activity between the two compounds [11].

In addition, the authors also compared the inhibition of MLA, aconitine (**30**), and lycoctonine (**2**) on ^3^H α-BGTx binding to musca domestica head homogenate. The rank order of inhibition is MLA > lycoctonine > aconitine, whereas the rank order of these alkaloids on nicotine receptors in rats is aconitine > MLA > lycoctonine. From the perspective of the mechanism of action, binding sites of these three compounds to muscle tissue and insect nerve tissue in mammals are different. MLA had strong inhibitory activity for the binding of ^3^H α-BGTx (α-buragarstoxin) to housefly head tissue, which suggested that MLA (**1**) may be a kind of invertebrates’ nicotinic acetylcholine receptor inhibitor. This provides some ideas for developing new insecticides.

**Figure 5:**
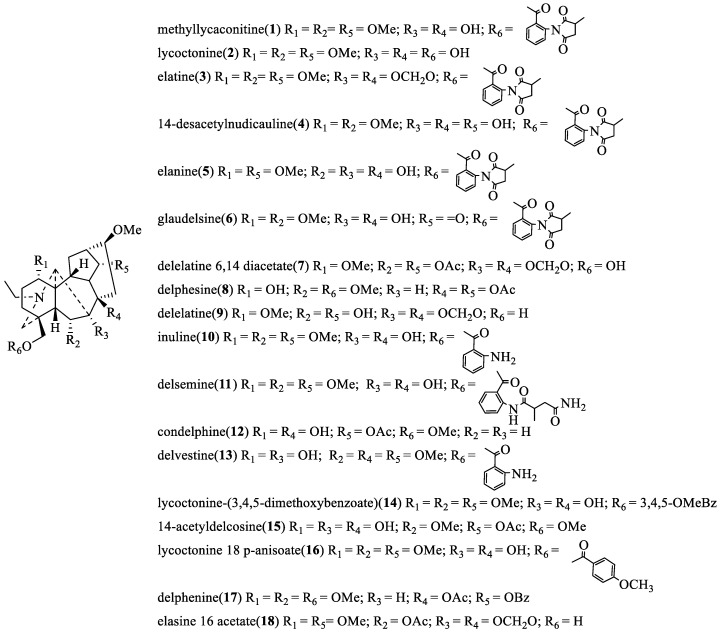

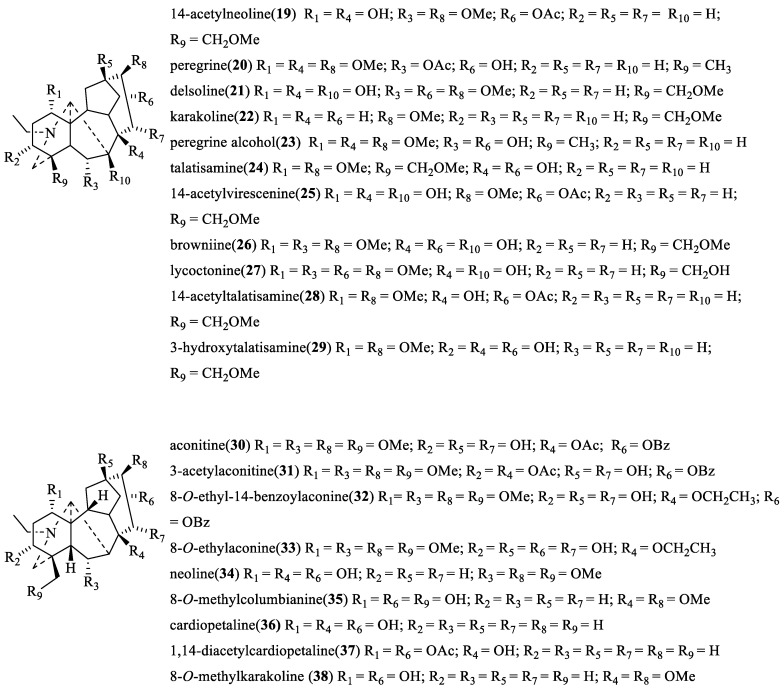

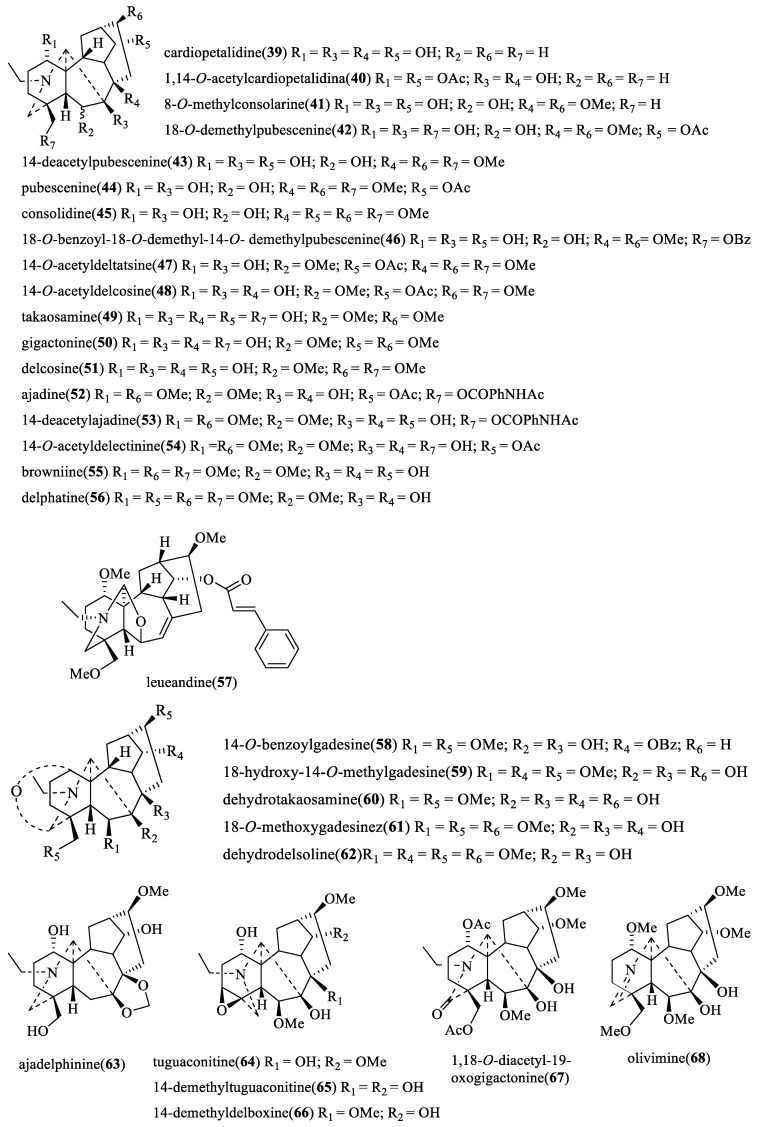

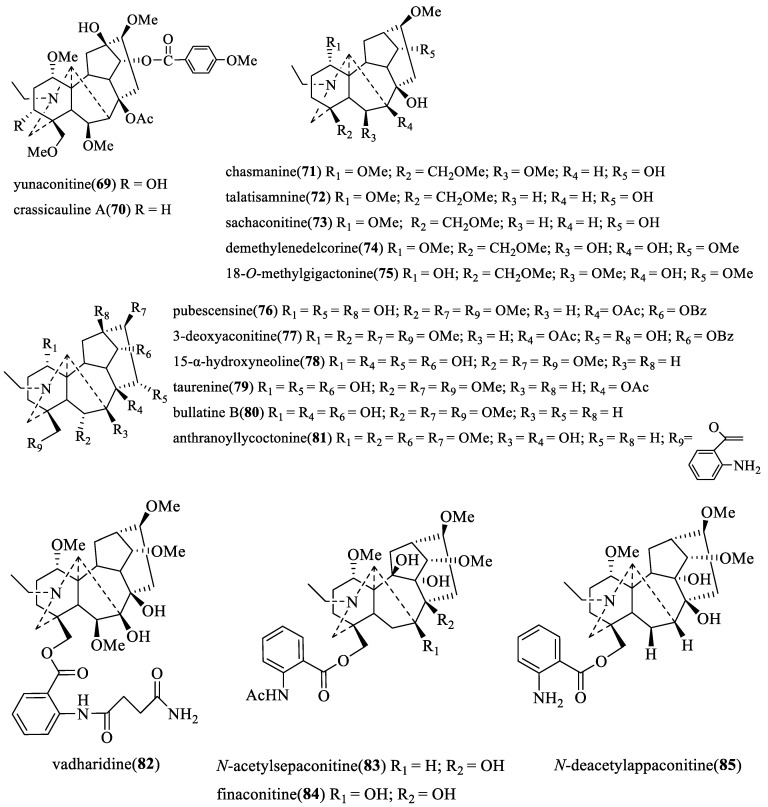

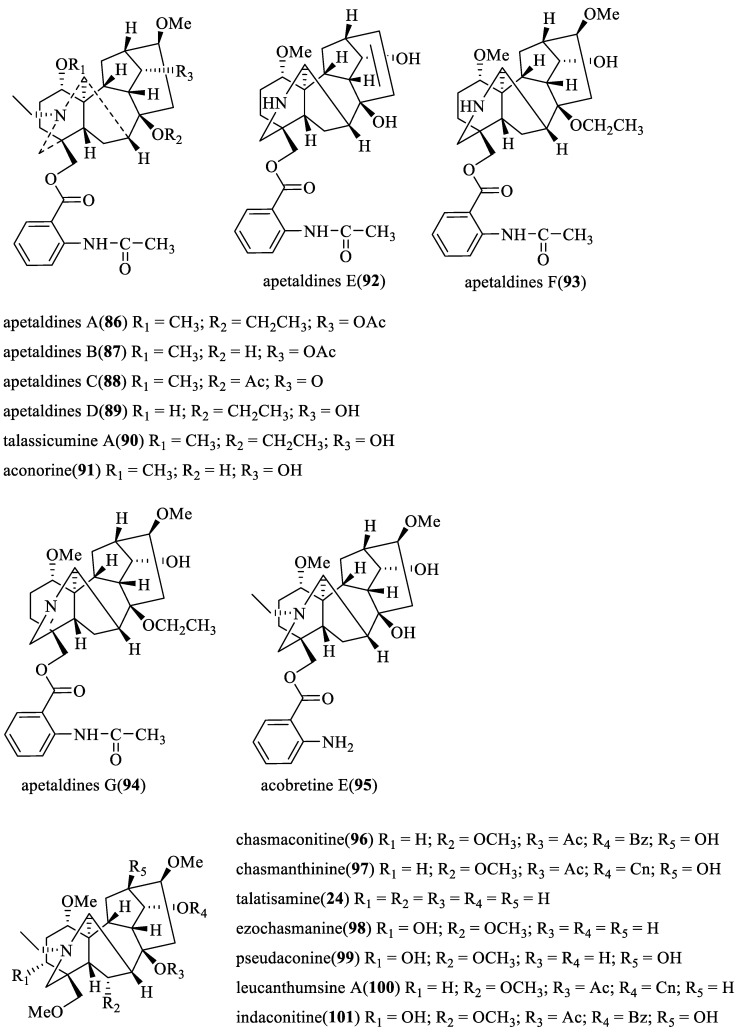

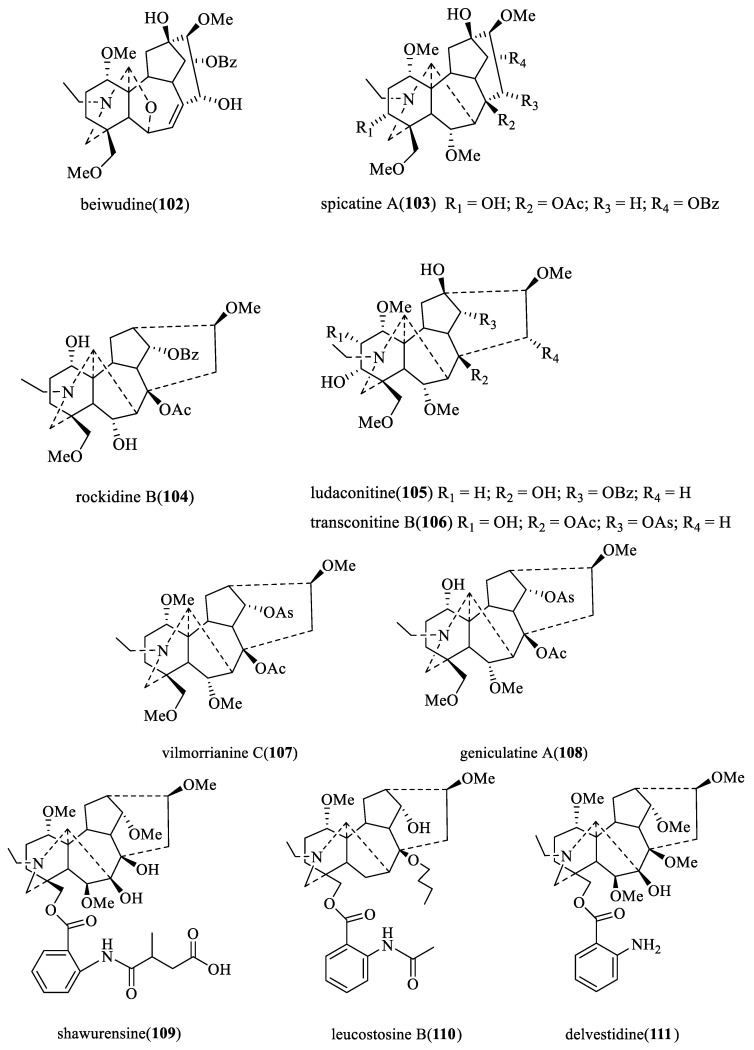
The structural formula of C_19_-diterpenoid alkaloids.

Since then, several diterpenoid alkaloids and their bioinsecticidal activities have been reported. Seventeen C_19_-diterpenoid alkaloids (compounds **1**, **3**–**18** in Figure 5) purified from *D.* spp. showed inhibitory activity of α-BGTx binding to rat and house fly neural membranes [12], which confirmed the earlier finding that C_19_-diterpenoid alkaloids may be inhibitors (Jennings et al. 1986) of α-BGTx [13]. The most potent inhibitory alkaloids tested in this series possessed the succinimide aromatic ester moiety in the C-18 position of MLA. Glaudelsine (6) could be a candidate for insecticide development based on its potency and selectivity as a ligand of the insect nicotinic receptor, where the IC_50_ value of the acetylcholine receptor is 42 pM in house flies and 16 nM in rat. In 2001, twelve C_19_-diterpenoid alkaloids (compounds **12**, **19**–**25**, **55,** and **27**–**29** in Figure 5) were isolated from *Delphinium*, *Aconitum,* and *Consolida* species, and the repellent activity against *Tribolium casteneum* (*Herbst*.) was tested (values of repellency ranged from 34.37% to 53.12%, Table 1). All 21 compounds showed promising insect-repellent activity; the highest level of repellency was found in 14-acetylneoline (**19**), peregrine (**20**), and 3-hydroxytalatisamine (**29**) with 53.12%, while talatisamine (**24**) had a low repellent effect (34.37%) on the test insect species [14].

González-Coloma’s research team investigated the insect antifeedant and toxic activity of 43 C_19_-diterpenoid alkaloids on *S. littoralis* and *L. decemlineata* [15]. Most of these compounds were purified from *Delphinium*, *Aconitum*, and *Consolida* species, while compounds 1, 14-*O*-diacetylcardiopetaline (**40**), 14-Deacetylpubescenine (**43**), and 1, 18-*O*-diacetyl-19-oxo-gigactonine (**67**) were semi-synthesized from cardiopetaline (**36**), cardiopetalidine (**39**), and gigactonine (**50**), respectively. The most active antifeedants against *L. decemlineatas* were 1, 14-diacetylcardiopetaline (**37**), and 18-hydroxy-14-*O*-methylgadesine (**59**) with EC_50_ values < 0.2 μg/cm^2^, followed by 8-*O*-methylconsolarine (**41**), 14-*O*-acetyldelectinine (**54**), karakoline (**22**), cardiopetaline (**36**), 18-*O*-demethylpubescenine (**42**), 14-*O*-acetyldeltatsine (**47**), takaosamine (**49**), ajadine (**52**), and 8-*O*-methylcolumbianine (**35**) (EC_50_ < 1 μg/cm^2^). Compounds **52**, **47**, 14-*O*-acetyldelcosine (**48**), and delphatine (**56**) (EC_50_ < 3 μg/cm^2^) showed the strongest antifeedant activity to *S. littoralis*. None of the measured compounds affected the feeding behavior of these insects. The most toxic compound to *L. decemlineata*, among those tested, was aconitine (**30**), followed by cardiopetalidine (**39**) (mortality > 60%), 14-deacetylpubescenine (**43**), 18-*O*-benzoyl-18-*O*-demethyl-14-*O*-deacetyl-pubescenine (**46**), 14-*O*-acetyldelcosine (**48**), 14-deacetylajadine (**53**), and MLA (**1**) (mortality > 45%). Orally injected *S. littoralis* larvae were negatively affected by aconitine (**30**), cardiopetaline (**36**), cardiopetalidine (**39**), 1,14-*O*-acetylcardiopetalidina (**40**), 8-*O*-methylconsolarine (**41**), 14-deacetylpubescenine (**43**), 1,18-*O*-diacetyl-19-oxo-gigactonine (**67**), and olivemine (**68**), as well as eserine to varying degrees. The toxicity of these compounds on two biological models lacking neurotransmission [Spodoptera frugiperda cells (Sf9 cells) and Chinese hamster ovary cells (CHO cells)] and *Trypanosoma cruzi* epimastigotes were also determined. Only a few compounds exhibited selective cytotoxic effects against insect-derived Sf9 cells, such as 14-deacetylpubescenine (**43**), tuguaconitine (**64**), 14-demethyldelboxine (**66**), 14-*O*-acetyldelcosine (**48**), dehydrodelsoline (**62**), 18-*O*-demethylpubescenine (**42**), 1,18-*O*-diacetyl-19-oxo-gigactonine (**67**), 1,14-diacetylcardiopetaline (**37**), and delcosine (**51**). And none of these compounds was cytotoxic to mammalian CHO cells or *T. cruzi*., indicating that the cytotoxic mode of action is not neurotoxic, and the selectivity between insect and mammalian cells might be related to membrane factors. Neither the antifeedant nor the toxic activity of these compounds studied followed the expected SAR (structure–activity relationship) based on their receptor binding activity. In addition, their antifeedant effects did not correlate with toxicity, and there is no evidence of the direct link between antifeedant effects and antagonistic action of compounds on insect nAChRs.

In 2007, the same research team mentioned above tested these 43 C_19_-norditerpenoid alkaloids for their insecticidal effects (antifeedant and toxic) toward *T. cruzi* and *Leishmania infantum* and cytotoxicity against several tumoral cell lines (CT26, SW480, HeLa, SkMel25, and SkMel28) [16]. It was found that none of the tested C_19_-diterpenoid alkaloids showed insecticidal activity on the two parasites. Compounds such as neoline (**34**), pubescenine (**44**), 14-deacetylajadine (**53**), lycoctonine (**29**), dehydrotakaosamine (**60**), and ajadelphinine (**63**) exhibited irreversible cytotoxic effects on the tested cell lines. Furthermore, none of these active compounds had ester bonds at C-14 or C-18, which are important parameters for mammalian toxicity [17].

Found in *A. episcopale*, four compounds including chasmanine (**71**), talatisamine (**24**), karakoline (**10**), and sachaconitine (**73**) showed obvious antifeedant effects on *Tribolium castaneum*, with EC_50_ values of 297.0, 342.8, 395.3, and 427.8 ppm, respectively [18]. Compared with two other diterpenoid alkaloids, yunaconitine (**69**) and crassicauline A (**70**), which lacked antifeedant activities, active compounds have a C-14 hydroxy group in their structure, which may influence the antifeedant activity of the compounds. Two C_19_-diterpenoid alkaloids, demethylenedelcorine (**74**) and 18-*O*-methylgigactonine (**75**), have been further isolated and identified from *A. sinomontanum* Nakai by bioassay-guided method; they have been proven to possess antifeeding and mortality effects on *Mythimna separata*.

In 2014, together with pubescensine (**76**), five known diterpenoid alkaloids, including 3-deoxyaconitine (**77**), aconitine (**30**), 15-α-hydroxyneoline (**78**), taurenine (**79**), and bullatine B (**80**), were isolated from the roots of *A. soongaricum* by Zhou Xianli and their team, and exhibited antifeedant activities against *Pieris rapae* Linne [19]. The highest antifeedant activities were found for compounds aconitine, pubescensine, and 3-deoxyaconitine (EC_50_ < 0.05 mg/cm^2^), respectively, followed by bullatine B, 15-α-hydroxyneoline, and taurenine (EC_50_ < 1 mg/cm^2^). Five natural diterpenoid alkaloids were further isolated and purified from *A. leucostomum* Vorosch [19]. Compounds anthranoyllycoctonine (**81**) and avadharidine (**82**) showed potent antifeedant activity (EC_50_ < 1 mg/cm^2^), followed by *N*-acetylsepaconitine (**83**), finaconitine (**84**), and *N*-deacetylappaconitine (**85**) (EC_50_ < 2 mg/cm^2^). In 2017, they investigated the antifeedant effects of 20 diterpenoid alkaloids against *S. exigua* for their ongoing search for natural products-based pesticides from genus *Delphinium* and *Aconitum* [20]. Among the compounds tested, chasmanthinine (**97**) showed highly potent antifeedant activity with an effective concentration for 50% feeding reduction (EC_50_) at 0.07 mg/cm^2^. Compounds apetaldines A (**86**), apetaldines E (**92**), chasmaconitine (**96**), leucanthumsine A (**100**), and indaconitine (**101**) also displayed higher potencies (EC_50_ values were 0.45, 0.28, 0.20, 0.18, and 0.41 mg/cm^2^) than those of compounds apetaldines B (**87**), apetaldines D (**89**), apetaldines F (**93**), talassicumine A (**90**), and aacobretine E (**95**) (EC_50_ values of 0.94, 0.64, 0.68, 0.76, and 0.66 mg/cm^2^). The structure–activity study of the antifeedant action of the test alkaloids showed that an amine (−NH−) moiety, Δ15(16) double bond, an anthranilic acid scaffold at C-18, esterification of HO-8 and/or HO-14, a cinnamoyl group at C-14, and oxygenation at C-13 strengthen the antifeedant potency.

In 2018, Shan Lianhai et al. isolated 19 kinds of diterpenoid alkaloids from *Delphinium naviculare var. lasiocarpum*, and evaluated the antifeedant activity of most of them against *S*. *exigua* [21]. The results showed that shawurensine (**109**) had the highest antifeedant activity with an EC_50_ of 0.45 mg/cm^2^ in the choice test and 0.81 mg/cm^2^ in the no-choice test. In the choice test, methyllycaconitine (**1**) and lappaconitine (**145**) (EC_50_ < 1 mg/cm^2^) showed the highest antifeedant activity. The antifeedant activities of the compounds shawurensine (**109**) and methyllycaconitine (**1**) compared with lycoctonine (**27**) indicated that substituting with an anthranilic acid scaffold at C-18 may improve the activity. In 2019, thirteen aconitine-type C_19_-diterpenoid alkaloids, two 7,17-subschizoid C_19_-diterpenoid alkaloids, and one lappaconine-type C_18_-diterpenoid alkaloid were isolated from *Aconitum karakolicum* Rapaics by Shan et al. [22]. The antifeedant activities of five C_19_-diterpenoid alkaloids have been tested, and the results showed that the aconitine (**30**) had a strong antifeedant activity with an EC_50_ of 0.02 mg/cm^2^ against the third-instar larvae of *Spodoptera exigua*. 3-deoxyaconitine (**77**), indaconitine (**101**), and beiwudine (**102**) also exhibited high antifeedant effects (EC_50_ < 2 mg/cm^2^).

In 2021, Ren Jiali and her team isolated a number of diterpenoid alkaloids from *Aconitum rockii Fletcher et Lauener* and tested their antifeedant activity against the third-instar larvae of *S. exigua* by leaf disc test [23]. The test results showed that 26 monomer compounds were shown in the C_19_-diterpenoid rejection activity test. These compounds have a certain level of food rejection activity. Among them, the most active compounds are rockidine B (**104**), ludaconitine (**105**), vilmorrianine C (**107**), indaconitine (**101**), transconitine B (**106**), yunaconitine (**69**), and geniculatine A (**108**), and EC_50_ values are 0.32, 0.77, 0.68, 0.27, 0.29, 0.35, and 0.35 mg/cm^2^, respectively. Combined with the analysis of the structure of the compound, it can be seen that the hydroxyl group of position 3 and the benzene-containing cyclic substituent of position 14 can enhance the non-eating activity of the compound. If the rejection activity of rockidine B is lower than that of yunaconitine (**69**), the hydroxyl group of position 3 also improves the activity of the compound to a certain extent; the hydroxyl substitution of position 13 can also enhance the rejection activity of the compound. When comparing yunaconitine (**69**) and geniculatine A (**108**), it is evident that both compounds exhibit strong rejection activity, suggesting that the hydroxyl substitution at position 13 can also enhance the activity. The benzene ring substitution of position 14 has a great impact on the compound’s rejection activity, which can enhance the rejection activity of the compound.

In the same year, Jue Wang and her team isolated several compounds from *Aconitum leucostomum Worosch* [24] and tested their antifeedant activity against *S*. *exigua* using a selective leaf disc test. The results showed that leucostosine B (**110**) and delvestidine (**111**) had the strongest antifeedant activity, with EC_50_ of 1.54 mg/cm^2^ and 2.82 mg/cm^2^, respectively.

#### 2.2.2. Insecticidal and Inhibiting Activities of C_20_-Ditepenoid Alkaloids

**Figure 6:**
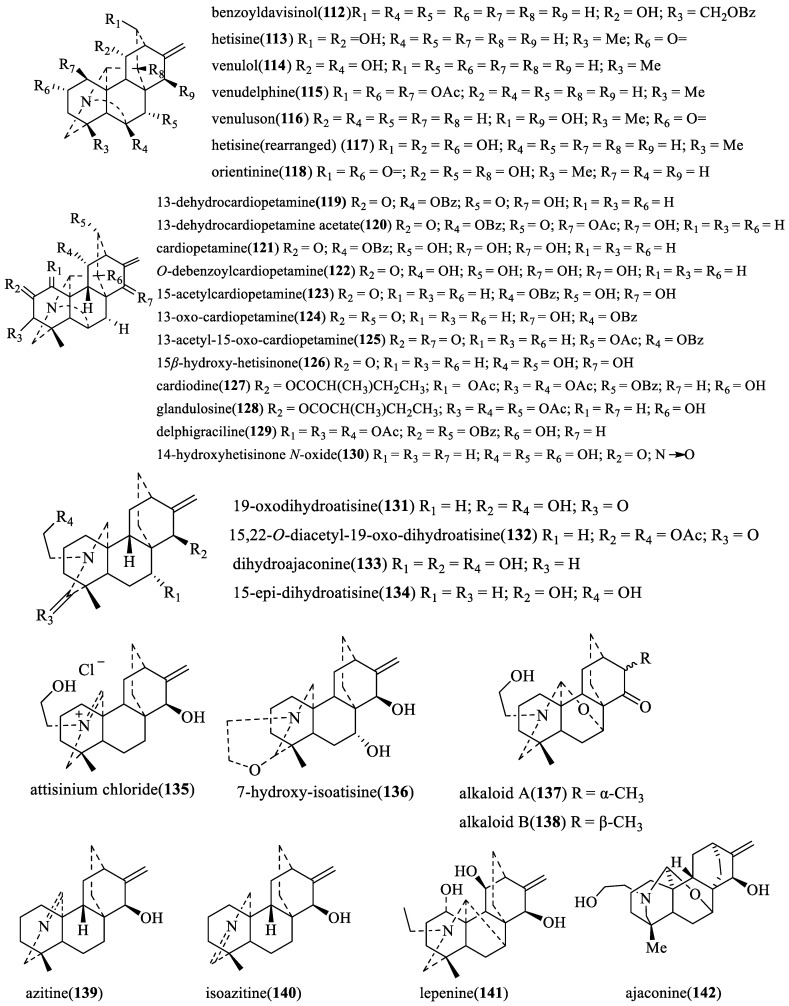
The structural formula of C_20_-diterpenoid alkaloids.

In 2001, the repellent activity of six C_20_-diterpenoid alkaloids (compounds **112**–**117** in Figure 6), isolated from *Delphinium*, *Aconitum*, and *Consolida* species, against *T. casteneum* (Herbst.) was tested (Table 2) [14]. The highest level of repellency was found in hetisine (59.12%), while venulol had a low repellent effect (31.25%) against the test insect species.

The test on insect antifeedant and toxic activity of C_20_-diterpenoid alkaloids (compounds **119**–**123** in Figure 6), conducted by González-Coloma’s research team, indicated that none of these compounds show toxicity against *S. littoralis* and *L. decemlineata* [25]. Cardiopetamine (**121**) and 15-acetylcardiopetamine (**123**) significantly inhibited the feeding activity of *S. littoralis* and *L. decemlineata*, suggesting a potential broad range of antifeedant action. The hydroxyl substitution of C-13 and C-15 was essential for the activity on *S. littoralis*, while the hydroxyl acetylation of C-13 and C-15 had positive effects on the antifeeding activity against *L. decemlineata*. The benzyl group of C-11 showed positive effect on both insect species. Furthermore, cardiopetamine did not show antifungal activity, and it did not cause any mutagenic effects on *Salmonella* strains, indicating that this alkaloid does not have general toxic and genotoxic effects.

In 2004, the same research team tested the insect antifeedant and toxic activity of 21 C_20_-diterpenoid alkaloids (compounds **113**, **121**, **123**–**128**, **131**–**140**, and **117** in Figure 6) from *Delphinium*, *Aconitum,* and *Consolida species* on *S. littoralis* and *L. decemlineata* [26]. Their toxicity on the two biological models lacking neurotransmission [Spodoptera frugiperda cells (Sf9 cells) and Chinese hamster ovary cells (CHO cells)] and their antiparasitic effects against *Trypanososma cruzi* and *Leishmania infantum* were also tested. *S. littoralis* had a stronger response to the active compounds than *L. decemlineata* (EC_50_ values ranging between 2 and 28 and from 0.1 to 24 μg/cm^2^ for *L. decemlineata* and *S. littoralis*, respectively). Additionally, the antifeedant effects of the DAs tested on CPB were lower than those previously reported for NDAs (with EC_50_ values for NDAs on CPB ranging between 0.1 and 12 μg/cm^2^) [14]. 19-oxodihydroatisine (**131**, EC_50_ = 0.1 μg/cm^2^) and the rearranged form of hetisine(rearranged) (**117**, EC_50_ = 1.7 μg/cm^2^) were the most active antifeedants to *S. littoralis* and *L. decemlineata*, respectively. Only glandulosine (**128**) was slightly toxic to *S. littoralis* larvae. A few compounds—13-oxo-cardiopetamine (**124**), 19-oxo-dihydroatisine (**131**), and atisinium chloride (**135**)—showed cytotoxic effects to insect-derived Sf9 with varying degrees of selectivity with respect to mammalian CHO cells. Compounds 13-oxo-cardiopetamine (**124**) and 15,22-*O*-diacetyl-19-oxodihydroatisine (**132**) had a toxic effect on *Trypanosoma cruzi*.

In 2007, the research team further determined the insecticidal activity of 22 C_20_-diterpenoid alkaloids against *Trypanosoma cruzi*, *Spodopteralittoralis* and *Leptinotarsa decemlineata*. 19-Oxodihydroatisine (**131**), 15,22-*O*-diacetyl-19-oxo-dihydroatisine (**132**), azitine (**139**), and isoazitine (**140**) possessed high insecticidal activity against *Spodoptera littoralis and Leptinotarsa decemlineata*, while 13-oxo-cardiopetamine (**124**) and atisinium chloride (**135**) had good insecticidal effects on *Trypanosoma cruzi.*

One study by M. Reina (2007) determined the insecticidal activity of delphigraciline (**129**) and 14-hydroxyhetisinone-*N*-oxide (**130**) against *T. cruzi* and *L. protozoa* [27]. Delphigraciline (**129**) possessed insecticidal activity against *T. cruzi* (IC_50_ = 7.3 µg/mL) in vitro, which is similar with the reported insecticidal effect atisine-type diterpenoid alkaloids [28], and 14-hydroxyhetisinone *N*-oxide (**130**) exhibiting trace insecticidal effect on both parasites. Concurrent studies showed that compound **130** also did not display any antifeedant activity. Previously, it was reported that 15-hydroxyhetisinone, an analog of compound **130**, had antifeedant effects on *Spodoptera littoralis*, indicating that this type of compound had specific antifeedant activity against *S. litura.*

In 2012, Chun-Lan Yuan et al. examined the insecticidal and antifeedant activities of lepenine (**141**) from *A. sinomontanum* Nakai against *M. separata* [29]. Interestingly, the results showed that the feeding stimulation of the compound was strong, and its rate reached 99.5% at 72 h.

#### 2.2.3. Insecticidal and Inhibiting Activities of C_18_-Ditepenoid Alkaloids

**Figure 7:**
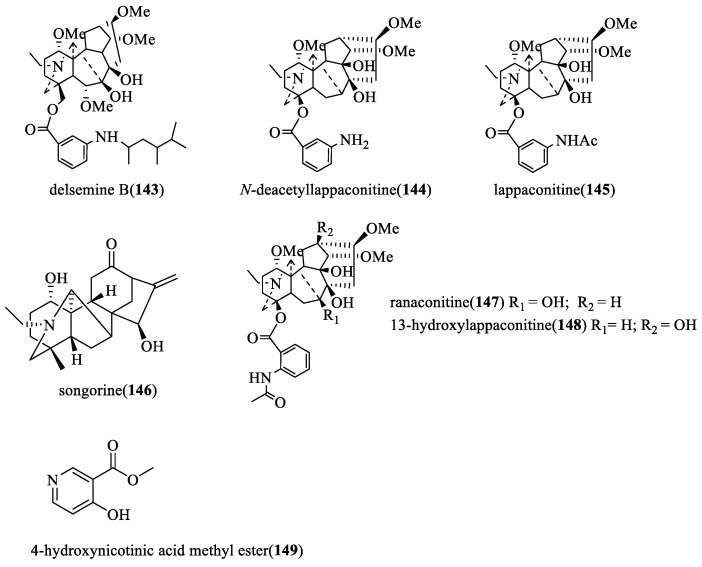
The structural formula of C_18_-diterpenoid alkaloids.

In 2001, three C_18_-diterpenoid alkaloids (**143**, **144**, and **145** in Figure 7) were isolated from *Delphinium*, *Aconitum*, and *Consolida* species, and the repellent activity against *Tribolium casteneum* (*Herbst.*) was tested. *N*-deacetyllappaconitine (**143**) of them showed good repellent activity (50.00%) [14].

In 2020, the contact toxicity of three C_18_-diterpenoid alkaloids and 4-hydroxynicotinic acid methyl ester (**149**), isolated from *Aconitum anthoroideum* DC., against *Nilaparvata lugens* Stal and *Sogatella furcifera* Horvath was tested [30]. The results showed that the four compounds had significant contact activity, and ranaconitine (**147**) exhibited the strongest insecticidal activity with the LD_50_ of 0.26 μg/insect and 0.25 μg/insect after 48 h treatment, respectively.

## 3. Summary

We searched for the biological activities of diterpene alkaloids in the genera Aconitum and Delphinium through databases such as SciFinder, Web of Science, and CNKI. Meanwhile, we screened, analyzed, and organized the data by establishing tables.

As shown in Table 3, diterpenoid alkaloids, as secondary metabolites of plants, show varying degrees of insecticidal and antifeedant activity against a variety of insects. Diterpenoid alkaloids are diverse in species and structures, and the screening of low-toxic and high-efficiency plant pesticides has broad prospects. However, there are still several problems in the study of insect biological effects of these compounds. Firstly, although there is a lot of previous research on the activity of diterpenoid alkaloids involving both types (C_19_ and C_20_) and a small number of studies on the C_18_, none of those deal with the activity of bis-diterpenoid alkaloids. Considering that different types of compounds may have different activities on the same insect, the biological activities of bis-diterpenoid alkaloids should also be studied. The same compound may exhibit different biological activities on different kinds of insects. So, it is necessary to expand the selection of tested insect species. Thirdly, the research on the insecticidal and antifeedant activity of diterpenoid alkaloids is not systematic enough. Many activity studies only focus on a few alkaloids isolated from a certain plant, which leads to a lack of sufficient data to support the activity comparison between compounds of the same type. This may be because researchers prioritize compounds with known potential activity because of funding, technology, or time, thereby reducing risk and increasing efficiency. Except for the research group led by González-Coloma, which simultaneously investigated the antifeedant activities of 44 C_19_-diterpenoid alkaloids and 22 C_20_-diterpenoid alkaloids against *Spodoptera litura* and *Leptinotarsa decemlineata*, other activity studies are relatively scattered and not systematic. Therefore, when comparing the biological activity of the same type of compound, the evidence is slightly insufficient. Fourthly, using live worms for activity tests requires many samples, is time-consuming and laborious, and thus cannot achieve high-throughput and effective screening. In addition, many compounds are derived from natural products and have low content, which makes it difficult to conduct large-scale activity screening. Both reasons have led to insufficient data on the insect biological activity of DAs. Therefore, to quickly and effectively screen compounds, we should also consider combining advanced computer-assisted technology or using cell screening methods. Additionally, we can also use synthetic methods to modify its structure to obtain some safer compounds with better activity. Fifthly, research on the mechanism of DAs on insect biological defense is insufficient due to the lack of evidence of activity. Finally, given the different distribution of plant secondary metabolites in various plant tissues, further study is needed to determine whether the content of DAs or PAs in these tissues correlates with the strength of their insecticidal or antifeedant activity.

To advance this field, future research must adopt a holistic approach that integrates modern technologies to overcome these limitations. Combining multiple techniques can efficiently separate and identify compounds [31], thus enabling detailed assessment. Additionally, synthetic biology [32] and total synthesis methods [33] offer new approaches to increase the yield of diterpene alkaloids. Moreover, in total synthesis methods, the structure of diterpene alkaloids can be selectively modified to enhance their insecticidal effects and narrow the gap between natural product insecticides and actual pesticide development. By systematically addressing these knowledge gaps and leveraging interdisciplinary methodologies, the exploration of DAs can yield innovative, sustainable solutions for pest management, contributing to the global shift toward green agriculture and mitigating the risks of chemical pesticide resistance.

## Figures and Tables

**Table 1:** The average repellency of C_19_-diterpenoid alkaloids to *Triboliumm casteneum* [14].

Substance	Average Repellency(%)	Mean Repellency Class ^a^
condelphine	40.62	III
14-acetylneoline	53.12	III
peregrine	53.12	III
delsoline	37.50	II
karakoline	37.50	II
peregrine alcohol	37.50	II
talatisamine	34.37	III
14-acetylvirescenine	43.75	III
lycoctonine	46.87	III
14-acetyltalatisamine	46.87	III
3-hydroxytalatisamine	53.12	III
browniine	46.87	III

^a^ I: 0.1–20% (Repellency), II: 20.1–40%, III: 40.1–60%, IV: 60.1–80%, V: 80.1–100%.

**Table 2:** The average repellency of C_20_-diterpenoid alkaloids to *Triboliumm casteneum* [14].

Substance	Average Repellency(%)	Mean Repellency Class ^a^
benzoyldavisinol	46.87	III
hetisinone	37.50	II
venulol	31.25	II
ajaconine	53.12	III
venudelphine	40.62	III
venuluson	56.25	III
hetisine	59.12	III
orientinine	46.87	III

^a^ I: 0.1–20% (repellency), II: 20.1–40%, III: 40.1–60%, IV: 60.1–80%, V: 80.1–100%.

**Table 3:** Biological activity of diterpenoid alkaloids on insects/parasites/cells.

Compounds	Insect Species/Cells	Feeding *	Activity	**Relevant Data**	**Ref.**
methyllycaconitine (**1**)	*Spodoptera eridania*	C	Antifeedant activity	LC_50_ = 308 ppm	[10]
				feeding damage to the leaf was less than 5% at 100 ppm	
	*Musca domesticcs* (house fly)		Insecticidal activity	Active denotes significant (50% +) mortality at a screening rate of 1000 ppm	
	*Musca domesticcs*		Inhibition of α-Bungarotoxin	IC_50_ = 6.4 × 10^−10^ M	[12]
	Rat brain			IC_50_ = 1.7 × 10^−9^ M	
	--		Inhibition of ^3^H α-Bungarotoxin	Kinh = 0.25 ± 0.05 nM	[10]
aconitine (**30**)	--	-		Kinh = 2.7 ± 0.8 × 10^−4^ M	
lycoctonine (**2**)	--	-		Kinh = 3.8 ± 0.6 × 10^−7^ M	
elatine (**3**)	Rat brain	-	Inhibition of α-Bungarotoxin	IC_50_ = 4.3 ± 0.4 × 10^−9^ mol/L	[12]
	*Musca domestics* (house fly)	O		IC_50_ = 2.9 ± 0.1 × 10^−10^ mol/L	
14-desacetylnudicauline (**4**)	Rat brain	-		IC_50_ = 1.0 ± 0.1 × 10^−8^ mol/L	
	*Musca domesticcs*	O		IC_50_ = 8.8 × 10^−10^ mol/L	
elanine (**5**)	Rat brain	-		IC_50_ = 1.2 ± 0.4 × 10^−8^ mol/L	
	*Musca domesticcs*	O		IC_50_ = 1.1 ± 0.1 × 10^−8^ mol/L	
glaudelsine (**6**)	Rat brain	-		IC_50_ = 1.6 ± 0.7 × 10^−8^ mol/L	
	*Musca domesticcs*	O		IC_50_ = 4.2 ± 0.1 × 10^−11^ mol/L	
delelatine 6,14 diacetate (**7**)	Rat brain	-		IC_50_ = 8.9 ± 1.0 × 10^−8^ mol/L	
	*Musca domesticcs*	O		IC_50_ = 9.8 ± 0.1 × 10^−8^ mol/L	
delphesine (**8**)	Rat brain	-		IC_50_ = 1.4 ± 0.4 × 10^−7^ mol/L	
	*Musca domesticcs*	O		IC_50_ = 1.0 ± 0.1 × 10^−7^ mol/L	
delelatine (**9**)	Rat brain	-		IC_50_ = 2.9 ± 0.1 × 10^−7^ mol/L	
	*Musca domesticcs*	O		IC_50_ = 9.0 ± 0.1 × 10^−8^ mol/L	
anthranoyllycoctonine (inuline) (**10**)	Rat brain	-		IC_50_ = 3.4 ± 0.5 × 10^−7^ mol/L	
	*Musca domesticcs*	O		IC_50_ = 3.4 ± 0.1 × 10^−8^ mol/L	
delsemine (**11**)	Rat brain	-		IC_50_ = 3.6 ± 0.3 × 10^−7^ mol/L	
	*Musca domesticcs*	O		IC_50_ = 5.9 ± 0.5 × 10^−9^ mol/L	
condelphine (**12**)	*Tribolium casteneum*	S	Repellent activity	Average repellency = 40.63%	[14]
	Rat brain	-	Inhibition of α-Bungarotoxin	IC_50_ = 8.0 ± 1.1 × 10^−7^ mol/L	[12]
	*Musca domesticcs*	O		IC_50_ = 3.10 ± 0.01 × 10^−8^ mol/L	
delvestine (**13**)	Rat brain	-		IC_50_ = 1.6 ± 0.3 × 10^−6^ mol/L	
	*Musca domesticcs*	O		IC_50_ = 2.6 ± 0.4 × 10^−8^ mol/L	
lycoctonine-18-(3,4,5-dimethoxybenzoate (**14**)	Rat brain	-		IC_50_ = 2.8 ± 0.6 × 10^−6^ mol/L	
	*Musca domesticcs*	O		IC_50_ = 9.4 ± 0.6 × 10^−9^ mol/L	
14-acetyldelcosine (**15**)	Rat brain	-		IC_50_ = 4.9 ± 1.0 × 10^−6^ mol/L	
	*Musca domesticcs*	O		IC_50_ = 7.1 ± 0.4 × 10^−9^ mol/L	
lycoctonine 18 p-anisoate (**16**)	Rat brain	-		IC_50_ = 1.8 ± 0.3 × 10^−5^ mol/L	
	*Musca domesticcs*	O		IC_50_ = 3.0 ± 0.1 × 10^−8^ mol/L	
delphenine (**17**)				IC_50_ = 3.2 ± 0.1 × 10^−8^ mol/L	
elasine 16 acetate (**18**)	*Musca domesticcs*	O	Inhibition of α-Bungarotoxin	IC_50_ = 7.9 ± 0.2 × 10^−8^ mol/L	[12]
14-acetylneoline (**19**)	*Tribolium casteneum*	S	Repellent activity	Average repellency = 53.12%	[14]
peregrine (**20**)				Average repellency = 53.12%	
delsoline (**21**)				Average repellency = 37.50%	
karakoline (**22**)	*Leptinotarsa decemlineata*	C	Antifeedant activity	EC_50_ = 0.44 µg/cm^2^	[15]
	*Tribolium castaneum*	S	Antifeedant activity	EC_50_ = 395.3 ppm	[14]
			Repellent activity	Average repellency = 37.50%	
peregrine alcohol (**23**)				Average repellency = 37.50%	
talatisamine (**24**)				Average repellency = 34.37%	
14-acetylvirescenine (**25**)				Average repellency = 43.75%	
browniine (**55**)				Average repellency = 46.87%	
delsemine b (**143**)				Average repellency = 37.50%	
14-acetyltalatisamine (**28**)				Average repellency = 46.87%	
3-hydroxytalatisamine (**29**)				Average repellency = 53.12%	
*N*-deacetyllappaconitine (**144**)				Average repellency = 50.00%	
lappaconitine (**145**)				Average repellency = 34.37%	
lycoctonine (**27**)				Average repellency = 46.87%	
	SW480	-	Cytotoxicity	% V = 7 ± 2	[15]
aconitine (**30**)	*Spodoptera littoralis*	C	Insecticidal toxicity. A covariance analysis (ANCOV A1) of food consumption (ΔI) and biomass gains (ΔB) with initial larval weight as covariate, using oral injection.	ΔB = 34, ΔI = 67	
			Antifeedant activity	EC_50_ = 0.02 mg/cm^2^	
neoline (**34**)	SW480	-	Cytotoxicity	% V = 5 ± 0	
8-*O*-methylcolumbianine (**35**)	*Leptinotarsa decemlineata*	C	Antifeedant activity	EC_50_ = 0.99 µg/cm^2^	
	*Spodoptera littoralis*	C		EC_50_ > 50 µg/cm^2^	
cardiopetaline (**36**)	*Leptinotarsa decemlineata*	C	Antifeedant activity	EC_50_ = 0.42 µg/cm^2^	
			Insecticidal toxicity	% M = 4	
	*Spodoptera littoralis*	C	Insecticidal toxicity. A covariance analysis (ANCOV A1) of food consumption (ΔI) and biomass gains (ΔB) with initial larval weight as covariate, using oral injection.	ΔB = 26, ΔI = 70	
1,14-diactylcardiopetalina (**37**)	*Leptinotarsa decemlineata*	C	Antifeedant activity	EC_50_ = 0.11 µg/cm^2^	
cardiopetadine(**39**)	*Leptinotarsa decemlineata*	C	Insecticidal toxicity	% M = 61	
1,14-*O*-acetylcardiopetalidina(**40**)	*Spodoptera littoralis*	C	Insecticidal toxicity. A covariance analysis (ANCOV A1) of food consumption (ΔI) and biomass gains (ΔB) with initial larval weight as covariate, using oral injection.	ΔB = 69, ΔI = 112	[15]
8-*O*-methylconsolarine (**41**)	*Leptinotarsa decemlineata*	C	Antifeedant activity	EC_50_ = 0.23 µg/cm^2^	
	*Spodoptera littoralis*	C	Insecticidal toxicity. A covariance analysis (ANCOV A1) of food consumption (ΔI) and biomass gains (ΔB) with initial larval weight as covariate, using oral injection.	ΔB = 79, ΔI = 94	
18-*O*-demethylpubescenine (**42**)	*Leptinotarsa decemlineata*	C	Antifeedant activity	EC_50_ = 0.60 µg/cm^2^	
	SF9 cell	-	Cytotoxicity	LD_50_ = 29.17 µg/ml	
14-deacetyl-pubescenine (**43**)	*Leptinotarsa decemlineata*	C	Insecticidal toxicity	% M = 47	
	*Spodoptera littoralis*	C	Insecticidal toxicity. A covariance analysis (ANCOV A1) of food consumption (ΔI) and biomass gains (ΔB) with initial larval weight as covariate, using oral injection.	ΔB = 78, ΔI = 95	
	SF9 cell	-	Cytotoxicity	LD_50_ = 0.38 µg/ml	
pubescenine (**44**)	SW480	-	Cytotoxicity (determined with MTT method)	% V = 10 ± 0	
18-*O*-benzoyl-18-*O*-demethyl-14-*O*-demethylpubescenine (**46**)	*Leptinotarsa decemlineata*	C	Insecticidal toxicity	% M = 11	
14-*O*-acetyldeltatsine (**47**)	*Leptinotarsa decemlineata*	C	Antifeedant activity	EC_50_ = 0.54 µg/cm^2^	
	*Spodoptera littoralis*	C		EC_50_ = 0.84 µg/cm^2^	
14-*O*-acetyl-delcosine (**48**)	*Spodoptera littoralis*	C	Antifeedant activity	EC_50_ = 1.51 µg/cm^2^	
	*Leptinotarsa decemlineata*	C	Insecticidal toxicity	% M = 41	
	SF9 cell	-	Cytotoxicity	LD_50_ = 14.88 µg/ml	
takaosamine (**49**)	*Leptinotarsa decemlineata*	C	Antifeedant activity	EC_50_ = 0.66 µg/cm^2^	
delcosine (**51**)	SF9 cell	-	Cytotoxicity	LD_50_ = 32.37 µg/ml	
ajadine (**52**)	*Leptinotarsa decemlineata*	C	Antifeedant activity	EC_50_ = 0.84 µg/cm^2^	
14-deacetylajadine (**53**)	*Leptinotarsa decemlineata*	C	Insecticidal toxicity	% M = 47	
	SW480	-	Cytotoxicity (determined with MTT method)	Not enough compound available	
14-*O*-acetydelectinine (**54**)	*Leptinotarsa decemlineata*	C	Antifeedant activity	EC_50_ = 0.29 µg/cm^2^	
delphatine (**56**)	*Spodoptera littoralis*	C	Antifeedant activity	EC_50_ = 2.72 µg/cm^2^	
methyllycaconitine (**1**)	*Leptinotarsa decemlineata*	C	Insecticidal toxicity	% M = 47	[15]
18-hydroxy-14-*O*-methlygadesine (**59**)			Antifeedant activity	EC_50_ = 0.13 µg/cm^2^	
dehydrotakaosamine (**60**)	SW480	-	Cytotoxicity (determined with MTT method)	% V = 5 ± 0	
dehydrodelsoline (**62**)	SF9 cell	-	Cytotoxicity	LD_50_ = 18.89 µg/ml	
ajadelphinine (**63**)	SW480	-	Cytotoxicity (determined with MTT method)	% V = 4 ± 0	
tuguaconitine (**64**)	SF9 cell	-	Cytotoxicity	LD_50_ = 1.83 µg/ml	
14-demethyldelboxine (**66**)	SF9 cell	-	Cytotoxicity	LD_50_ = 6.27 µg/ml	
1,18-*O*-diacetyl-19-oxo-gigactonine (**67**)	*Spodoptera littoralis*	C	Insecticidal toxicity	EC_50_ > 50 µg/ml	
	SF9 cell	-	Cytotoxicity	LD_50_ = 29.45 µg/ml	
olivimine (**68**)	*Spodoptera littoralis*	C	Insecticidal toxicity	EC_50_ > 50 µg/cm^2^	
yunaconitine (**69**)	*Tribolium castaneum*	S	Antifeedant activity	EC_50_ = 653.4 ppm	[18]
crassicauline a (**70**)				EC_50_ = 1134.5 ppm	
chasmanine (**71**)				EC_50_ = 297.0 ppm	
talatisamnine (**72**)				EC_50_ = 342.8 ppm	
sachaconitine (**73**)				EC_50_ = 427.8 ppm	
demethylenedelcorine (**74**)	*mythimna separata*	C	Antifeedant activity, 72 h	% IR = 100	
			Insecticidal toxicity, 72 h	% M = 40.2	
18-*O*-methylgigactonine (**75**)	*mythimna separata*	C	Antifeedant activity, 72 h	% IR = 70.1	
			Insecticidal toxicity, 72 h	% M = 29.2	
pubescensine (**76**)	*Spodoptera littoralis*	C	Antifeedant activity	EC_50_ = 0.03 mg/cm^2^	[19]
3-deoxyaconitine (**77**)				EC_50_ = 0.05 mg/cm^2^	
15-α-hydroxyneoline (**78**)				EC_50_ = 0.47 mg/cm^2^	
taurenine (**79**)				EC_50_ = 0.66 mg/cm^2^	
bullatine b (**80**)				EC_50_ = 0.41 mg/cm^2^	
anthranoyllycoctonine (**81**)				EC_50_ = 0.73 mg/cm^2^	
avadharidine (**82**)				EC_50_ = 0.84 mg/cm^2^	
*N*-acetylsepaconitine (**83**)				EC_50_ = 1.21 mg/cm^2^	
finaconitine (**84**)				EC_50_ = 1.44 mg/cm^2^	
*N*-deacetylappaconitine (**85**)				EC_50_ = 1.88 mg/cm^2^	
apetaldines a (**86**)	*Spodoptera littoralis*	C	Antifeedant activity	EC_50_ = 0.45 mg/cm^2^	[20]
apetaldines b (**87**)				EC_50_ = 0.94 mg/cm^2^	
apetaldines c (**88**)				EC_50_ = 1.18 mg/cm^2^	
apetaldines d (**89**)				EC_50_ = 0.64 mg/cm^2^	
apetaldines e (**92**)				EC_50_ = 0.28 mg/cm^2^	
apetaldines f (**93**)				EC_50_ = 0.68 mg/cm^2^	
apetaldines g (**94**)				EC_50_ = 9.23 mg/cm^2^	
talassicumine a (**90**)				EC_50_ = 0.76 mg/cm^2^	
aconorine (**91**)				EC_50_ = 5.65 mg/cm^2^	
aacobretine e (**95**)				EC_50_ = 1.75 mg/cm^2^	
taurenine (**79**)	*Spodoptera littoralis*	C	Antifeedant activity	EC_50_ = 0.66 mg/cm^2^	[20]
songorine (**145**)				EC_50_ = 60 mg/cm^2^	
chasmaconitine (**96**)				EC_50_ = 0.2 mg/cm^2^	
chasmanthinine (**97**)				EC_50_ = 0.07 mg/cm^2^	
talatisamine (**24**)				EC_50_ = 50 mg/cm^2^	
ezochasmanine (**98**)				EC_50_ = 2.09 mg/cm^2^	
pseudaconine (**99**)				EC_50_ = 1.79 mg/cm^2^	
leucanthumsine a (**100**)				EC_50_ = 0.18 mg/cm^2^	
indaconitine (**101**)				EC_50_ = 0.41 mg/cm^2^	
leueandine (**57**)				EC_50_ = 3.32 mg/cm^2^	
benzoyldavisinol (**112**)	*Tribolium casteneum*	S	Repellent activity	Average repellency = 46.87%	[14]
hetisine (**113**)	*Spodoptera littoralis*	C	Antifeedant activity	EC_50_ > 50 µg/cm^2^	[26]
			Insecticidal toxicity. A covariance analysis (ANCOV A1) of food consumption (ΔI) and biomass gains (ΔB) with initial larval weight as covariate, using oral injection.	ΔB = 93.2, ΔI = 110.7	
	*Leptinotarsa decemlineata*	C	Antifeedant activity	EC_50_ = 13.1 µg/cm^2^	
	*Tribolium casteneum*	S	Repellent activity	Average repellency = 37.50%	[14]
venulol (**114**)	*Tribolium casteneum*	S	Repellent activity	Average repellency = 31.25%	
ajaconine (**142**)	*Spodoptera littoralis*	C	Antifeedant activity	EC_50_ = 8.2 µg/cm^2^	[26]
			Insecticidal toxicity. A covariance analysis (ANCOV A1) of food consumption (ΔI) and biomass gains (ΔB) with initial larval weight as covariate, using oral injection.	ΔB = 80.4, ΔI = 104.5	
	*Leptinotarsa decemlineata*	C	Antifeedant activity	EC_50_ = 5.1 µg/cm^2^	
	*Tribolium casteneum*	S	Repellent activity	Average repellency = 53.12%	[14]
venudelphine (**115**)				Average repellency = 40.62%	
venuluson (**116**)				Average repellency = 56.25%	
hetisine(rearranged) (**117**)	*Spodoptera littoralis*	C	Antifeedant activity	EC_50_ > 50 µg/cm^2^	[26]
			Insecticidal toxicity. A covariance analysis (ANCOV A1) of food consumption (ΔI) and biomass gains (ΔB) with initial larval weight as covariate, using oral injection.	ΔB = 89.5, ΔI = 121.9	
	*Leptinotarsa decemlineata*	C	Antifeedant activity	EC_50_ = 1.73 µg/cm^2^	
	*Tribolium casteneum*	S	Repellent activity	Average repellency = 59.12%	[14]
orientinine (**118**)				Average repellency = 46.87%	
15-acetylcardiopetamine (**123**)	*Leptinotarsa decemlineata*	C	Antifeedant activity	EC_50_ = 12.86 nmol/cm^2^	[25]
	*Spodoptera littoralis*	C	Antifeedant activity	EC_50_ > 100 nmol/cm^2^	
cardiopetamine (**121**)	*Spodoptera littoralis*	C	Antifeedant activity	EC_50_ = 5.5 µg/cm^2^	[25]
			Insecticidal toxicity. A covariance analysis (ANCOV A1) of food consumption (ΔI) and biomass gains (ΔB) with initial larval weight as covariate, using oral injection.	ΔB = 110.3, ΔI = 103.3	
	*Leptinotarsa decemlineata*	C	Antifeedant activity	EC_50_ = 22.5 µg/cm^2^	
13-oxo-cardiopetamine (**124**)	*Spodoptera littoralis*	C	Antifeedant activity	EC_50_ > 100 µg/cm^2^	[26]
			Insecticidal toxicity. A covariance analysis (ANCOV A1) of food consumption (ΔI) and biomass gains (ΔB) with initial larval weight as covariate, using oral injection.	ΔB = 105.8, ΔI = 97.2	
	*Leptinotarsa decemlineata*	C	Antifeedant activity	Not enough compound available	
13-acetyl-15-oxo-cardiopetamine (**125**)	*Spodoptera littoralis*	C	Antifeedant activity	EC_50_ > 100 µg/cm^2^	
			Insecticidal toxicity. A covariance analysis (ANCOV A1) of food consumption (ΔI) and biomass gains (ΔB) with initial larval weight as covariate, using oral injection.	ΔB = 87.9, ΔI = 113.9	
	*Leptinotarsa decemlineata*	C	Antifeedant activity	EC_50_ = 27.2 µg/cm^2^	
15β-hydorxy-hetisinone (**126**)	*Spodoptera littoralis*	C	Antifeedant activity	EC_50_ = 23.7 µg/cm^2^	
			Insecticidal toxicity. A covariance analysis (ANCOV A1) of food consumption (ΔI) and biomass gains (ΔB) with initial larval weight as covariate, using oral injection.	ΔB = 104.5, ΔI = 106.5	
cardiodine (**127**)	*Spodoptera littoralis*	C	Antifeedant activity	EC_50_ = 4.4 µg/cm^2^	
			Insecticidal toxicity. A covariance analysis (ANCOV A1) of food consumption (ΔI) and biomass gains (ΔB) with initial larval weight as covariate, using oral injection.	ΔB = 97.6, ΔI = 119.7	
	*Leptinotarsa decemlineata*	C	Insecticidal toxicity. A covariance analysis (ANCOV A1) of food consumption (ΔI) and biomass gains (ΔB) with initial larval weight as covariate, using oral injection.	EC_50_ = 2.2 µg/cm^2^	
glandulosine (**128**)	*Spodoptera littoralis*	C	Antifeedant activity	EC_50_ > 50 µg/cm^2^	
			Insecticidal toxicity. A covariance analysis (ANCOV A1) of food consumption (ΔI) and biomass gains (ΔB) with initial larval weight as covariate, using oral injection.	ΔB = 82.93, ΔI = 73.48	
glandulosine (**128**)	*Leptinotarsa decemlineata*	C	Antifeedant activity	EC_50_ = 4.0 µg/cm^2^	[26]
delphigraciline (**129**)	*Leptinotarsa decemlineata*	C	Antifeedant activity	EC_50_ = 12.2 µg/cm^2^	[27]
	*Trypanosoma cruzi*	-	Insecticidal toxicity	IC_50_ = 7.3 mg/ml	
19-oxodihydroatisine (**131**)	*Spodoptera littoralis*	C	Antifeedant activity	EC_50_ = 0.1 µg/cm^2^	[26]
	*Spodoptera littoralis*	C	Insecticidal toxicity. A covariance analysis (ANCOV A1) of food consumption (ΔI) and biomass gains (ΔB) with initial larval weight as covariate, using oral injection.	ΔB = 90, ΔI = 92	
	*Leptinotarsa decemlineata*	C	Antifeedant activity	EC_50_ > 50 µg/cm^2^	
15,22-*O*-diacetyl-19-oxo-dihydroatisine (**132**)	*Spodoptera littoralis*	C	Antifeedant activity	EC_50_ = 6.1 µg/cm^2^	
		-	Insecticidal toxicity. A covariance analysis (ANCOV A1) of food consumption (ΔI) and biomass gains (ΔB) with initial larval weight as covariate, using oral injection.	ΔB = 91, ΔI = 85	
	*Leptinotarsa decemlineata*	C	Antifeedant activity	EC_50_ > 50 µg/cm^2^	
dihydroajaconine (**133**)	*Spodoptera littoralis*	C	Antifeedant activity	EC_50_ > 50 µg/cm^2^	
			Insecticidal toxicity. A covariance analysis (ANCOV A1) of food consumption (ΔI) and biomass gains (ΔB) with initial larval weight as covariate, using oral injection.	ΔB = 80.5, ΔI = 97.2	
	*Leptinotarsa decemlineata*	C	Antifeedant activity	EC_50_ = 5.0 µg/cm^2^	
15-epi-dihydroatisine (**134**)	*Spodoptera littoralis*	C	Antifeedant activity	EC_50_ > 50 µg/cm^2^	
			Insecticidal toxicity. A covariance analysis (ANCOV A1) of food consumption (ΔI) and biomass gains (ΔB) with initial larval weight as covariate, using oral injection.	ΔB = 98.10, ΔI = 96.37	
	*Leptinotarsa decemlineata*	C	Antifeedant activity	EC_50_ = 2.9 µg/cm^2^	
attisinium chloride (**135**)	*Spodoptera littoralis*	S	Antifeedant activity	EC_50_ = 2.4 µg/cm^2^	
			Insecticidal toxicity. A covariance analysis (ANCOV A1) of food consumption (ΔI) and biomass gains (ΔB) with initial larval weight as covariate, using oral injection.	ΔB = 123, ΔI = 103	
	*Leptinotarsa decemlineata*	C	Antifeedant activity	EC_50_ = 3.4 µg/cm^2^	
7-hydroxy-isoatisine (**136**)	*Spodoptera littoralis*	C	Antifeedant activity	EC_50_ > 50 µg/cm^2^	[26]
			Insecticidal toxicity. A covariance analysis (ANCOV A1) of food consumption (ΔI) and biomass gains (ΔB) with initial larval weight as covariate, using oral injection.	ΔB = 93.6, ΔI = 119.2	
	*Leptinotarsa decemlineata*	C	Antifeedant activity	EC_50_ = 3.4 µg/cm^2^	
alkaloid a (**137**)	*Spodoptera littoralis*	C	Antifeedant activity	EC_50_ = 50 µg/cm^2^	
			Insecticidal toxicity. A covariance analysis (ANCOV A1) of food consumption (ΔI) and biomass gains (ΔB) with initial larval weight as covariate, using oral injection.	ΔB = 112.5, ΔI = 115.4	
	*Leptinotarsa decemlineata*	C	Antifeedant activity	EC_50_ = 5.4 µg/cm^2^	
alkaloid b (**138**)	*Spodoptera littoralis*	C	Antifeedant activity	EC_50_ > 50 µg/cm^2^	
			Insecticidal toxicity. A covariance analysis (ANCOV A1) of food consumption (ΔI) and biomass gains (ΔB) with initial larval weight as covariate, using oral injection.	ΔB = 101.0, ΔI = 119.9	
	*Leptinotarsa decemlineata*	C	Antifeedant activity	EC_50_ = 3.6 µg/cm^2^	
azitine (**139**)	*Spodoptera littoralis*	C	Antifeedant activity	EC_50_ = 1.1 µg/cm^2^	
			Insecticidal toxicity. A covariance analysis (ANCOV A1) of food consumption (ΔI) and biomass gains (ΔB) with initial larval weight as covariate, using oral injection.	ΔB = 109, ΔI = 99	
	*Leptinotarsa decemlineata*	C	Antifeedant activity	EC_50_ > 50 µg/cm^2^	
isozitine (**140**)	*Spodoptera littoralis*	C	Antifeedant activity	EC_50_ = 4.1 µg/cm^2^	
			Insecticidal toxicity. A covariance analysis (ANCOV A1) of food consumption (ΔI) and biomass gains (ΔB) with initial larval weight as covariate, using oral injection.	ΔB = 115, ΔI = 100	
	*Leptinotarsa decemlineata*	C	Antifeedant activity	EC_50_ = 6.9 µg/cm^2^	
beiwudine (**102**)	*Spodoptera littoralis*	C	Antifeedant activity	EC_50_ = 1.81 mg/cm^2^	[22]
spicatine a (**103**)				EC_50_ = 8.18 mg/cm^2^	
rockidine b (**104**)				EC_50_ = 0.32 mg/cm^2^	[23]
ludaconitine (**105**)				EC_50_ = 0.77 mg/cm^2^	
vilmorrianine c (**107**)				EC_50_ = 0.68 mg/cm^2^	
transconitine b (**106**)				EC_50_ = 0.29 mg/cm^2^	
geniculatine a (**108**)				EC_50_ = 0.35 mg/cm^2^	
4-hydroxynicotinic acid methyl ester (**149**)	*Nilaparvata lugens*	C	Contact toxicity	LD_50_ = 0.33 ± 0.05 μg/insect	[30]
	*Sogatella furcifera*	C		LD_50_ = 0.26 ± 0.03 μg/insect	
ranaconitine (**146**)	*Nilaparvata lugens*	C		LD_50_ = 0.26 ± 0.03 μg/insect	
	*Sogatella furcifera*	C		LD_50_ = 0.25 ± 0.02 μg/insect	
shawurensine (**109**)	*Spodoptera littoralis*	C	Antifeedant activity	EC_50_ = 0.45 mg/cm^2^	[21]
				EC_50_ = 0.81 mg/cm^2^	
leucostosineb (**110**)				EC_50_ = 1.54 mg/cm^2^	[24]
delvestidine (**111**)				EC_50_ = 2.82 mg/cm^2^	
13-hydroxylappaconitine (**148**)	*Nilaparvata lugens*	C	Contact toxicity	LD_50_ = 0.38 ± 0.05 μg/insect	[27]
	*Sogatella furcifera*	C		LD_50_ = 0.33 ± 0.02 μg/insect	

* Insects were classified as C—crop pests, S—stored product pests, O—others (incl. mites and termites).

## Data Availability

No new data were created or analyzed in this study.

## References

[1] Aronson A.I., Dunn P.E. (1991). Biological Pesticide.

[2] Zhang J.F., Wang W., Lu X.H., Xu Y.Q., Zhang X.H. (2010). The stability and degradation of a new biological pesticide, pyoluteorin. Pest Manag. Sci..

[3] David P., Rajinder P. (1995). Integrated Pest Management.

[4] Makkar H.P.S., Siddhuraju P., Becker K., Clifton N.J. (2007). Plant Secondary Metabolites. Methods in Molecular Biology.

[5] Wang G.Q., Ji L.Z., Zhang H., Wang X.W. (2006). Current Progress in Research of Botanical Insecticides in China. Sci. Agric. Sin..

[6] Wang F.P. (2021). Retrospection on studies of diterpenoid alkaloidal chemistry. NPRD.

[7] Shen Y., Liang W.J., Shi Y.N., Kennelly E.J., Zhao D.K. (2020). Structural diversity, bioactivities, and biosynthesis of natural diterpenoid alkaloids. Nat. Prod. Rep..

[8] Wang F.P. (2009). Modern Chemistry of Natural Products.

[9] Manske R.H.F., Rodrigo R.G.A. (1979). The Alkaloids.

[10] Jennings K.R., Brown D.G., Wright D.P. (1986). Methyllycaconitine, a naturally occurring insecticide with a high affinity for the insect cholinergic receptor. Cell. Mol. Life Sci..

[11] Macallan D.R.E., Lunt G.G., Wonnacott S., Swanson K.L., Rapoport H., Albuquerque E.X. (1988). Methyllycaconitine and (+)-anatoxin-a differentiate between nicotinic receptors in vertebrate and invertebrate nervous systems. FEBS Lett..

[12] Kukel C.F., Jennings K.R. (1994). Delphinium alkaloids as inhibitors of α-bungarotoxin binding to rat and insect neural membranes. Can. J. Physiol. Pharmacol..

[13] Ward J.M., Cockcroft V.B., Lunt G.G., Smillie F.S., Wonnacott S. (1990). Methyllycaconitine: A selective probe for neuronal α-bungarotoxin binding sites. FEBS Lett..

[14] Ulubelen A., Meriçli A.H., Meriçli F., Kilinçer N., Ferizli A.G., Emekci M., Pelletier S.W. (2001). Insect repellent activity of diterpenoid alkaloids. Phytother. Res..

[15] Coloma A.G., Reina M., Medinaveitia A., Guadaño A., Santana O., Martínez-Díaz R., Ruiz-Mesía L., Alva A., Grandez M., Díaz R. (2004). Structural diversity and defensive properties of norditerpenoid alkaloids. J. Chem. Ecol..

[16] Reina M., Coloma A.G. (2007). Structural diversity and defensive properties of diterpenoid alkaloids. Phytochem. Rev..

[17] Ameri A. (1998). The effects of Aconitum alkaloids on the central nervous system. Prog. Neurobiol..

[18] Liu Z.L., Cao J.Z., Hai M., Lin L.L., Liu H.J., Du S.S., Zhou L., Deng Z.W. (2011). Feeding deterrents from *Aconitum episcopale* roots against the red flour beetle, *Tribolium castaneum*. J. Agric. Food Chem..

[19] Chen L. (2017). Studies on Akaloid Constituents of Four Medicinal Plants and Antifeedant Activities of *Spodoptera exigua* Hiibner. Ph.D. Thesis.

[20] Zhang J.F., Chen L., Huang S., Shan L.H., Gao F., Zhou X.L. (2017). Diterpenoid Alkaloids from Two Aconitum Species with Antifeedant Activity against Spodoptera exigua. J. Nat. Prod..

[21] Shan L.H., Chen L., Gao F., Zhou X.L. (2019). Diterpenoid alkaloids from Delphinium naviculare var. lasiocarpum with their antifeedant activity on Spodoptera exigua. Nat. Prod. Res..

[22] Shan L.H., Chen L., Zhou X.L. (2019). Diterpenoid alkaloids from Aconitum karakolicum Rapaics. Nat. Prod. Res..

[23] Ren J.L. (2021). Diterpenoid Alkaloids from *Aconitum rockii* and Their Antifeedant Activity. Ph. D. Thesis.

[24] Wang J. (2021). Isolationand Biologicalactivities Screening Ofdiiterpenealkaloids from Aconitum leucostomum Worosch. Ph. D. Thesis.

[25] González-Coloma A., Guadaño A., Gutiérrez C., Cabrera R., de la Peña E., de la Fuente G., Reina M. (1998). Antifeedant delphinium diterpenoid alkaloids. Structure-activity relationships. J. Agric. Food Chem..

[26] González-Coloma A., Reina M., Guadaño A., Martínez-Díaz R., Díaz J.G., García-Rodriguez J., Alva A., Grandez M. (2004). Antifeedant C_20_ diterpene alkaloids. Chem. Biodivers..

[27] Reina M., Mancha R., Gonzalez-Coloma A., Bailen M., Rodriguez M.L., Martinez-Diaz R.A. (2007). Diterpenoid alkaloids from *Delphinium gracile*. Nat. Prod. Res..

[28] González P., Marín C., Rodríguez-González I., Hitos A.B., Rosales M.J., Reina M., Díaz J.G., González-Coloma A., Sánchez-Moreno M. (2005). In vitro activity of C_20_-diterpenoid alkaloid derivatives in promastigotes and intracellular amastigotes of *Leishmania infantum*. Int. J. Antimicrob. Agents.

[29] Yuan C.L., Wang X.L. (2012). Isolation of active substances and bioactivity of *Aconitum sinomontanum* Nakai. Nat. Prod. Res..

[30] Fan D.L., Yang L.L., Zeng D.Q., Tang W.X. (2020). Insecticidal alkaloids constituents from *Aconitum anthoroideum* DC. J. Environ. Entom..

[31] Song S.Y., Lan X.M., Xu J., Cui Y.F., Zhou H.Y., Zheng J., Dai S.Y., Zhang J.Y. (2023). Systematic analysis and identification of diterpenoid alkaloids from *Aconitum carmichaeli* Debx. by UHPLC-Q-Exactive Orbitrap MS. J. Pharm. Anal..

[32] Wang X., Cao X.Q., Guo D.M., Xu F.R., Ma X.H. (2024). Cloning and Expression Analysis of Acetyl-CoA Acetyltransferase Gene from *Aconitum vilmorinianum*. Chin. Med. Mat..

[33] Li C., Zhang C., Liu X.Y., Qin Y. (2024). Recent Progress in the Total Synthesis of Diterpenoid Alkaloids. Chin. J. Org. Chem..

